# Tribology of the sock-skin Interface – the influence of different fabric parameters on sock friction

**DOI:** 10.1186/s13047-022-00560-5

**Published:** 2022-08-19

**Authors:** Ian J. DeBois, Esha Agarwal, Ashish Kapoor, Kavita Mathur

**Affiliations:** North Carolina State University Raleigh, Raleigh, NC United States

**Keywords:** Friction blisters, Socks, Knit structure, Fiber composition, Yarn type, Friction, Tribology

## Abstract

**Background:**

The purpose of this parametric design of experiments was to identify and summarize how the influence of knit structure (single jersey vs. terry), fiber composition (polyester vs. cotton), fiber linear density (30/1 Ne vs. 18/1 Ne & 1/150/34 vs. 2/150/34), and yarn type (filament vs. spun) affected the frictional profile across the sock-skin interface.

**Methods:**

Friction testing trials were completed against both a polypropylene probe and a synthetic skin material (Lorica soft®) to determine if there was a difference in friction based on interface interaction. Friction testing was completed by sliding a probe across the inside bottom surface of the sock (the part that is usually in-contact with the bottom of the foot) while instantaneously measuring the frictional force every tenth of a second.

**Results:**

For both trials (plastic probe and synthetic skin), in the dry condition, knit structure was found to be the most prominent fabric parameter affecting the frictional force experienced at the sock-skin interface. It was also determined that fiber linear density, and yarn type are tertiary factors affecting the frictional force measured at the sock-skin interface. Finally, in the dry state, it was determined that fiber composition had seemingly no effect on the frictional force experienced at the sock-skin interface.

**Conclusion:**

This parametric design of experiments has further enhanced the understanding of the tribology at the sock-skin interface. Through strategic design, four different textile parameters have been investigated, measured, and justified as to how each influence the friction measured between the two interfaces. This knowledge can be used to develop socks that mitigate the risk of friction blisters formation.

## Introduction

Friction blisters are frequently occurring painful injuries, common to the foot, that affect everyone from athletes and military personnel to any active individual. Friction blisters are debilitating and can often lead to significant lifestyle changes to reduce or cope with pain [1–9]. For athletes such as marathon runner’s, friction blisters incidence during any given race can be between 0.2–39% and the pain from these friction blisters could have an adverse effect on their performance [1]. For military personnel such as cadets in training, friction blisters occur at rates of 42% and if these trends continue past instruction into the field, it could impose situations that are immediately dangerous to their health due to pain limiting or negatively affecting the execution of a task or mission. As for any active individual, friction blisters can lead to setbacks from personal goals, objectives, or work while the body heals from injury.

Friction blisters are formed as a result of abrasion caused by the frictional forces applied directly to the skin’s top layer of the epidermis, the stratum corneum. The frictional force applied to the stratum corneum is transmitted through the stratum granulosum into the stratum spinosum of the epidermis causing micro tearing in between skin layers. These micro-tears, cause separation of the epidermis as the prickly stratum spinosum cells detach from the underlying dermis layer [2, 7, 10]. Consequently, this results in a pocket of skin that fills with plasma-like fluid due to hydrostatic pressure [2, 7].

Several different associated factors have been conclusively studied, and have been shown to have a positive correlation with the occurrence of friction blister formation. These factors include: the influence of moisture or skin hydration on frictional force, the influence of normal force on frictional force, as well as the number of impact cycles the frictional force occurs over [3, 7, 8, 10–13] . Also, a person’s skin characteristics have been shown to contribute to the formation of friction blisters [10].

Looking past human and environmental influences, an area that is still unclear in regard to friction force experienced at the sock-skin interface is the role of different textile parameters. When reviewing published articles examining the effect of different fabric characteristics on friction blisters formation; contradicting findings, paired with inconsistencies between experimental procedures make comparing and determining conclusive results between studies fundamentally difficult. In the past, researchers have used a variety of different methods to characterize frictional force causing blister formation. Some studies have modified other friction measurement instruments to characterize fabric friction [2–5]. One study created a “custom built” frictional measuring device [6], while other studies have focused on field investigations where friction blister occurrence is analyzed and indirectly related back to frictional force [13, 14].

Based on the inconsistent findings between previous studies, the role of knit structure, fiber composition, and yarn linear density and type with regards to their influence on friction at the sock-skin interface have yet to be determined. In this parametric study, twelve common sock variations were strategically designed to better investigate these four different fabric parameters influence on the frictional force seen at this interface. Further, both a plastic probe and a human skin simulant (Lorica soft®) were analyzed to understand the difference in interface interaction based on contact material.

## Materials and methods

### Sample design

A design of experiments was developed based on a thorough analysis of commercially available socks as well as an industry expert’s advice. The sample design for this study involved twelve different sock variations examining two different knit structures (single jersey vs. terry). A single jersey knit, also referred to as a plain knit, is an interlooping structure where one row of needles creates a single thickness fabric where both sides are flat. A terry knit structure is a two-sided structure where one side resembles the flat nature of a single jersey knit fabric while the opposite side is characterized by soft piles or “terries”. Additionally, two different fiber composition (100% cotton vs. 100% polyester), with a high and low linear density (30/1 Ne vs. 18/1 Ne and their filament equivalents 1/150/34 and 2/150/34 respectively), and two different yarn types (filament vs. spun) as shown in Table 1 were investigated. The difference between filament and spun yarn is derived during their creation. Filament yarns are created by extruding one continuous yarn whereas spun yarns are created by twisting short staple fibers together to create one longer yarn. All socks were manufactured by experts at Manufacturing Solutions Center (Conover, NC), on a Lonati GK616DF cylindrical knitting machine. The needle count on the cylinder was 168, the gauge was 14 and the cylinder size was 95.25 mm. Further, all socks were plaited with 20/1/22/20 nylon and instructions were given to keep other variables such as tensioning, stitch setting, and cross-stretch as consistent as possible across all samples only changing these parameters when absolutely necessary.

**Table 1:** Sample design

Sample	Fabric Code	Fiber	Knit	Linear Density (Den)	Yarn Type	Thickness (mm)
**1**	30/1 CF	Cotton	Single Jersey	High*	Spun	5.19
**2**	30/1 CT	Cotton	Terry	High*	Spun	2.33
**3**	18/1 CF	Cotton	Single Jersey	Low**	Spun	2.51
**4**	18/1 CT	Cotton	Terry	Low**	Spun	6.03
**5**	1/150/34 PF	Polyester	Single Jersey	High*	Filament	2.11
**6**	1/150/34 PT	Polyester	Terry	High*	Filament	6.38
**7**	2/150/34 PF	Polyester	Single Jersey	Low**	Filament	2.47
**8**	2/150/34 PT	Polyester	Terry	Low**	Filament	5.70
**9**	30/1 PF	Polyester	Single Jersey	High*	Spun	2.39
**10**	30/1 PT	Polyester	Terry	High*	Spun	5.14
**11**	18/1 PF	Polyester	Single Jersey	Low**	Spun	2.46
**12**	18/1 PT	Polyester	Terry	Low**	Spun	6.38

*High linear density corresponds to 30 Ne for spun yarn and 1/150/34 for filament fibers

**Low linear density corresponds to 18 Ne for spun yarn and 2/150/34 for filament fibers

After receiving the socks from Manufacturing Solutions, they were then washed according to AATCC LP1–2018e using standard – normal washing and drying conditions outlined in the LP1–2018 procedure. Following washing and drying, the socks were laid flat in a standard conditioning room (23 +/− 1 °C; RH 50 +/− 2%) for 48 hours. The washing, drying, and conditioning process ensured dimensional stability within each variation and also created a more realistic condition of a sock a person may wear. After conditioning, each sock was labeled 1 through 5 then set aside for friction testing. Labelling was done to ensure socks were organized and tested without bias. A sixth sock of each variation was then imaged under a Keyence VHX7000 digital microscope at 50x magnification.

### Friction testing

**Fig. 1:**
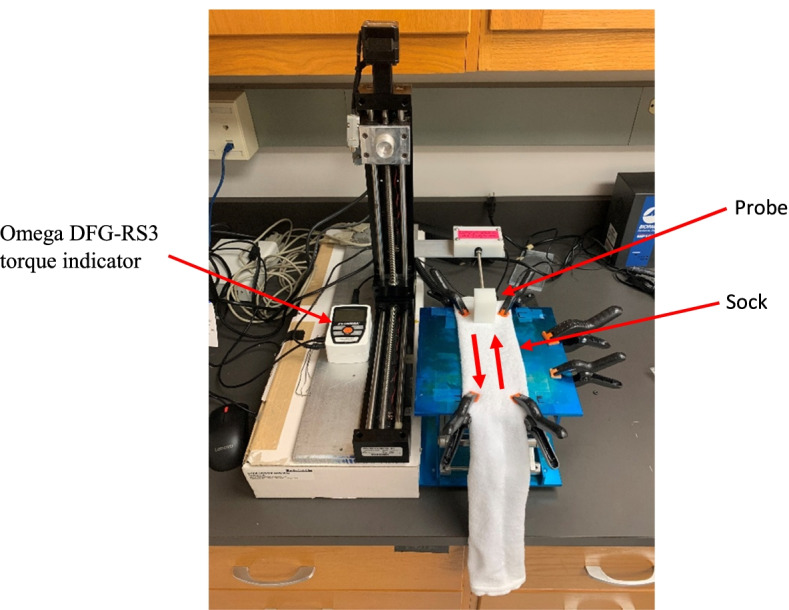
Custom friction measuring device

Two different sets of friction testing were completed. The first set of testing was a sock against a plastic probe integrated with an Omega DFG-RS3 torque indicator. The plastic probe was attached to a metal rod and the rod was attached to a stand that moved along a screw by way of electric motor. This setup allowed for measuring friction in two directions (out and back; represented by an arrow in Fig. 1) along the axis of a sock which a person’s foot would primarily slide along while wearing a sock. The plastic probe had a contact area of 30 mm × 30 mm. and was flat along all edges. A force of 60 N was applied by the probe to each sock creating a contact pressure of approximately 66 kPa similar to that of an average sized male. Force applied to the sock was measured using a single tact 15 mm diameter 45–450 N calibrated force sensor, connected to the computer by USB. All testing was conducted in a dry state.

Before testing began, socks were flipped inside out so the bottom inside of the sock was in contact with the probe. This is the surface that is usually in contact with the bottom of the foot when wearing a sock. During each test the probe cycled out and back twice, each cycle had a displacement of 127 mm in each direction and moved at a rate of 0.19 mm per second. Friction measurements were then taken every tenth of a second throughout the entire test by the Omega DFG-RS3 torque indicator. The friction measurements were recorded by the software MesurLite, and the raw data were was then exported to an Excel spreadsheet to be analyzed. In total each test cycle lasted 38.3 seconds and took 383 friction measurements.

During the second set of testing, all protocol remained the same but instead of the interface being between the probe and the sock, a small piece of Lorica soft skin simulant (49% polyurethane, 49% polyamide microfiber, 2% other fabric) was attached to the bottom of the probe. This created a sock – “skin-like” interface. Lorica was chosen as the skin simulant to create a more realistic interface based on its known use as an accurate simulant of human skin in a dry condition [15]. In both trials, all socks were secured to a platform via clamps to ensure no macroscale stretching or movement of the sock. Finally, the socks were stretched only enough to ensure no wrinkles, irregularities, or deformities were present at the interface as these could have impacted the results.

### Statistical analysis

Once all data had been recorded it was organized into two different excel spreadsheets based on the interface (probe vs. sock and Lorica vs. sock). The data were then further organized based on sliding direction in order to discriminate the knit’s orientations affect on friction. Once the data were organized, it was graphed as measured frictional force vs. sliding distance to understand the trends or irregularities within the data. Due to the senstivity of the force sensor, occasionally single points at discrete intervals were observed to have large jumps in measured frictional force. These points where subjected to further testing but were found to be noise from the torque indicator. Therefore, these data were normalized using the average of the previous and superseding points. This method was deemed appropriate based on the torque indicator’s buffer system where it takes an average of the surrounding points to calculate a frictional force. Once all the data had been normalized it was subjected to a one-way (univariate) ANOVA test to see if there was a statistically significant difference between socks. Once statistical significance between socks was confirmed, a multiple comparisons analysis completed to better understand which fabric parameters provided statistically different results. In total, 66 comparisons were made between each unique sock variation where each fabric parameter was directly compared. The results of the statistical analysis were found to be aligned with results of the raw data and can be seen Tables 2 and 3. Count is the number of readings made by the torque indicator over one test cycle. 384 is noted as the count because each test started at a frictional force of 0 before making 383 measurements while the probe was in motion. Average is the mean frictional force recorded by the torque indicator over the course of all tests for each sock variation. Sum is the count multipled by the average and is used to gauge the average total frictional force experienced over the entire test by the torque indicator. In this statistical analysis, variance was taken with respect to the average frictional force.

**Table 2:** Statistical analysis against lorica skin simulant

*Groups*	*Count*	*Sum*	*Average*	*Variance*
1–150-34 Poly Single Jersey Knit	384	70.17900	0.18276	0.00055
1–150-34 Poly Terry Knit	384	133.93700	0.34879	0.01181
2–150-34 Poly Single Jersey Knit	384	71.80800	0.18700	0.00075
2–150-34 Poly Terry Knit	384	125.00200	0.32553	0.01417
18–1 Cotton Single Jersey Knit	384	104.53100	0.27222	0.00293
18–1 Cotton Terry Knit	384	152.78600	0.39788	0.00626
30–1 Cotton Single Jersey Knit	384	90.70100	0.23620	0.00335
30–1 Cotton Terry Knit	384	197.32800	0.51388	0.00462
18–1 Poly Single Jersey Knit	384	80.65500	0.21004	0.00085
18–1 Poly Terry Knit	384	119.82100	0.31203	0.00398
30–1 Poly Single Jersey Knit	384	93.38100	0.24318	0.00258
30–1 Poly Terry Knit	384	199.73300	0.52014	0.00231

**Table 3:** Statistical analysis against probe

*Groups*	*Count*	*Sum*	*Average*	*Variance*
1–150-34 Poly Single Jersey Knit	384	43.49800	0.11328	0.00019
1–150-34 Poly Terry Knit	384	112.47700	0.29291	0.00324
2–150-34 Poly Single Jersey Knit	384	43.39700	0.11301	0.00015
2–150-34 Poly Terry Knit	384	78.91050	0.20550	0.00057
18–1 Cotton Single Jersey Knit	384	53.74700	0.13997	0.00047
18–1 Cotton Terry Knit	384	122.24600	0.31835	0.00368
30–1 Cotton Single Jersey Knit	384	61.29500	0.15962	0.00090
30–1 Cotton Terry Knit	384	167.47100	0.43612	0.00799
18–1 Poly Single Jersey Knit	384	55.23000	0.14383	0.00021
18–1 Poly Terry Knit	384	121.93050	0.31753	0.00136
30–1 Poly Single Jersey Knit	384	62.04900	0.16159	0.00055
30–1 Poly Terry Knit	384	179.83850	0.46833	0.02166

The effective difference measured in average frictional force and variance between Tables 2 and 3 can be attributed to the difference in interface interaction seen between the lorica skin simulant and the polypropylene probe. On a microlevel the polypropylene surface has less surface roughness and fewer defects than the lorica skin simulant. Therefore, the reduced interface interaction can be attributed for the lower average frictional force seen in the trials against the probe.

## Results

Each graph displays the instantaneously measured dynamic (sliding) friction along the inside bottom of the sock. Times were converted to displacement, then the profiles where graphed from 0 mm. to 127 mm and back 127 mm to 0 mm. When above the x-axis the probe was sliding in the out direction (0 mm – 127 mm), whereas when the values are negative the probe was sliding in the back direction (127mm – 0 mm). Therefore, the negative sign in front of the frictional forces measured below the x-axis denotes the probes sliding direction while the absolute value of that number would denote the measured force. Further, the force of static friction had to be overcome when the probe began movement and changed directions so it was expected that highest measured friction values in each graph occurred around the direction change then decreased.

### Friction testing with plastic probe

**Fig. 2:**
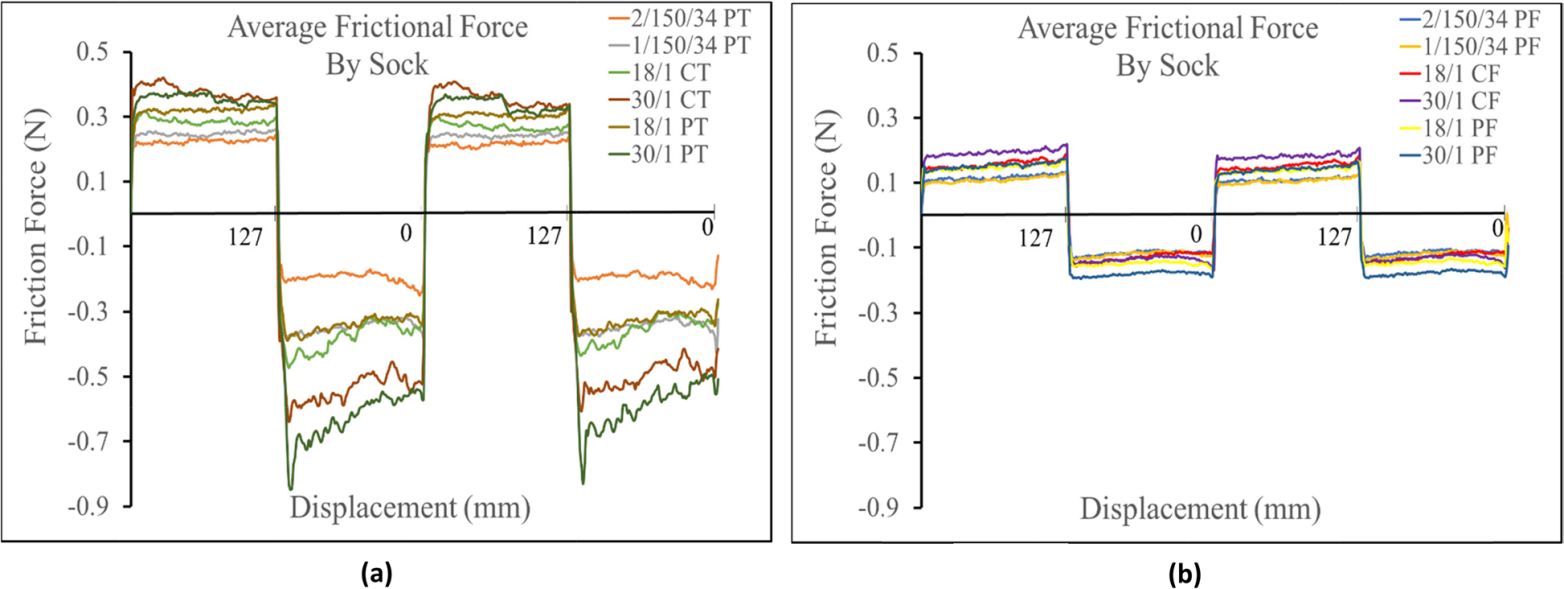
**a** Friction profiles comparing terry knit structures. **b** Friction profile comparing single jersey knit structures

The prominate determing factor in frictional force against the probe was found to be the socks knit structure. It was observed that terry knit structures always produced a higher frictional force than their corresponding single jersey knit structures (i.e. 30/1 PT > 301 PF). This trend can be seen in Fig. 2 where the six highest average frictional forces in both the positive and negative direction where all measured against terry knit fabrics. The corresponding single jersey knit structures all measured a lower frictional force and all had a very similar friction profile in both directions across each sock variation. The terry knit fabrics also showed a more sporadic friction profile along the socks primary sliding axis, (especially in the negative direction) than the single jersey knit strucutures. Figure 2 shows the average frictional profile across each sock comparing terry and single jersey knit structures.

**Fig. 3:**
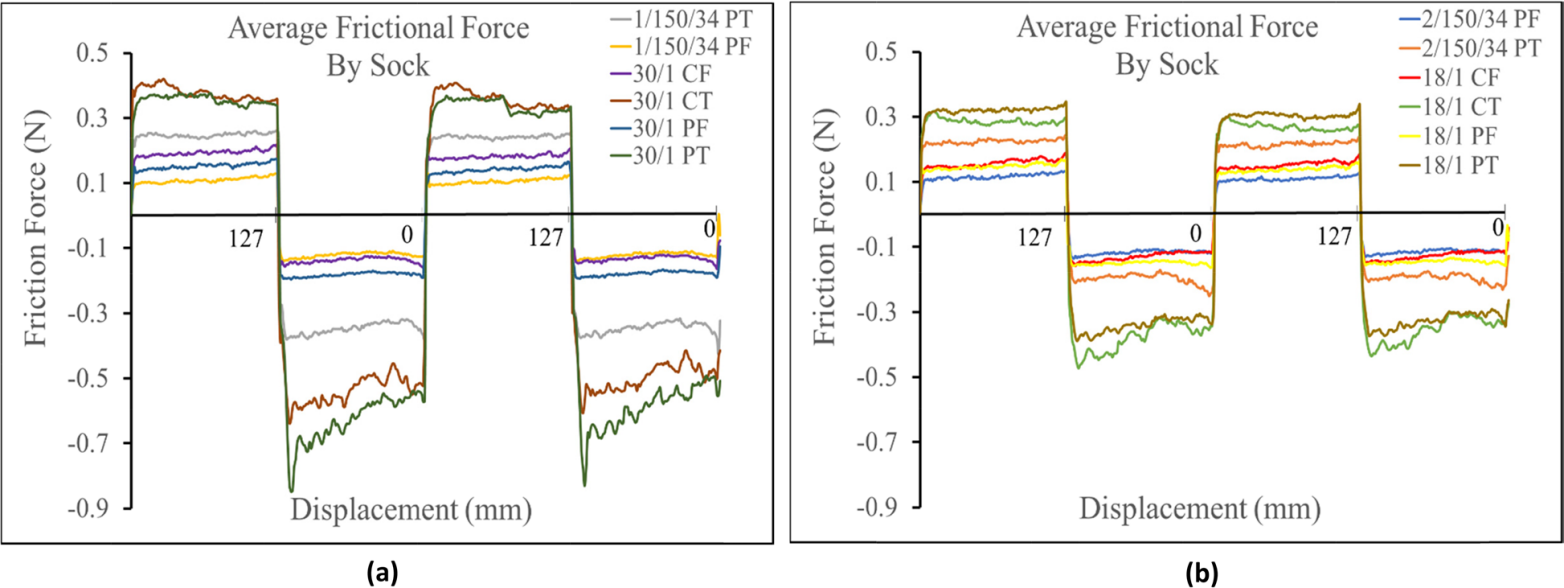
**a** Linear density comparison of terry knit samples against probe. **b** Linear density comparison of single jersey knit samples against probe

It was found that for both spun and filament yarns with terry knit structures, linear density was a secondary factor that influenced frictional force. All terry knit 30/1 Ne fibers produced a higher frictional force in both directions, than their corresponding terry knit socks made from the 18/1 Ne fibers (i.e. 30/1 PT > 18/1 PT and 30/1 CT > 18/1 CT). When comparing filament knit socks in the positive direction the 1/150/34 (30/1 Ne equivalent) filament yarns measured similar friction force to the 2/150/34 (18/1 Ne equivalent) across the socks profile. However, in the negative direction, the 1/150/34 filament sock measured a higher frictional force than the 2/150/34 (18/1 Ne equivalent). When comparing the frictional force of spun and filament single jersey knit strucutures based on linear density, there was almost no difference across their profiles as the frictional force measured for all sock variations was almost identical in both directions. Figure 3a shows the linear density comparison of the different sock structures for the terry knit samples. Figure 3b shows the linear density comparison for all sock variations of the single jersey knit samples.

**Fig. 4:**
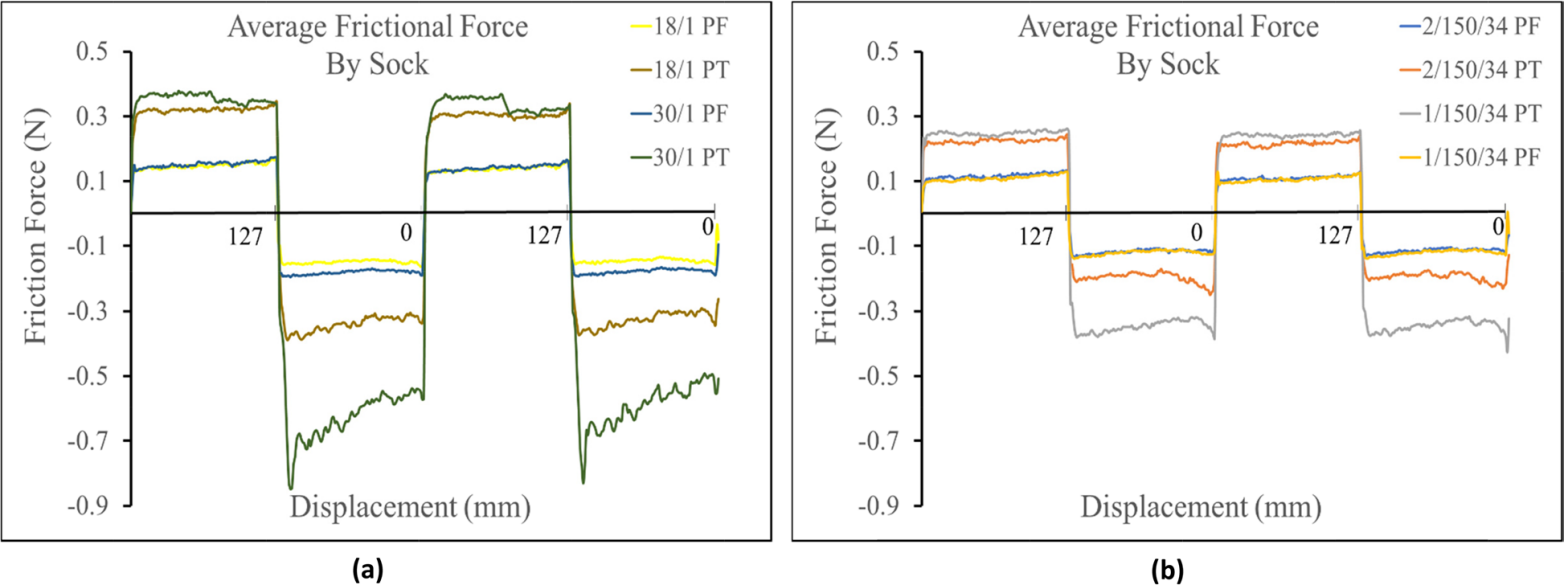
**a** Comparison of terry and single jersey knit spun polyester yarn. **b** Comparison of the equivalent terry and single jersey knit polyester filament yarn

Yarn type also was found to be a secondary factor that affected the frictional force measured across all sock profiles. For both terry and single jersey knit structures, the socks made from spun polyester yarns consistently produced a higher frictional force than the socks made from their corresponding polyester filament yarn samples. With the frictional force measuring higher in both directions. (i.e. 30/1 PT > 1/150/34 PT and 18/1 PT > 2/150/34 PT, 30/1 PF > 1/150/34 PF and 18/1 PF > 2/150/34 PF). Figure 4 shows the results when comparing spun polyester and polyester filament samples.

**Fig. 5:**
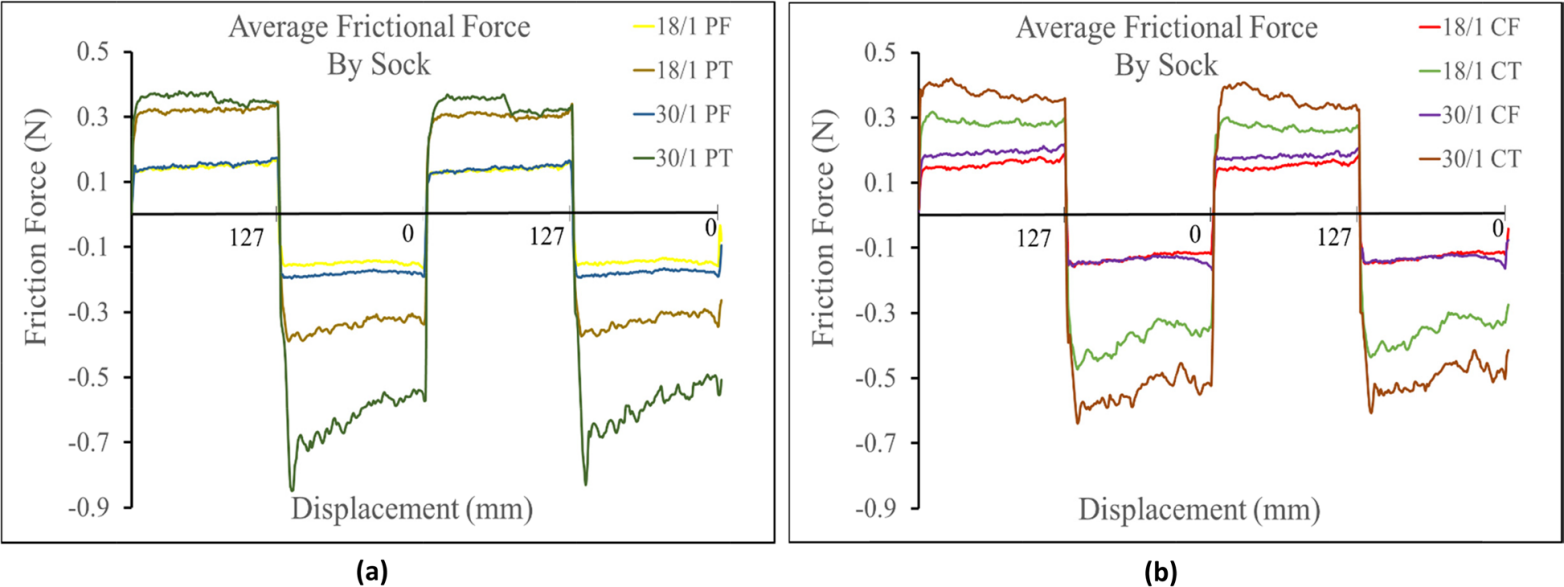
(**a**) Comparison of polyester fiber based on corresponding fabric structure, yarn type and linear density (**b**) Comparison of cotton fiber based on corresponding fabric structure, yarn type and linear density

Fiber composition did not seem to play a significant role in the frictional force measured against the probe. For the terry knit samples in the positive direction, the highest and lowest observed frictional forces were from the 30/1 and 18/1 cotton socks, respectively. While in the negative direction, the highest and lowest frictional forces measured were the 30/1 polyester and 18/1 polyester socks, respectively. Yet again, all the single jersey knit samples had a similar friction profile in the positive direction, with the 30/1 cotton single jersey knit measuring the highest frictional force in the positive direction. In the negative direction, once more, all the single jersey knit samples had a very similar friction profile, with the 30/1 polyester samples registering the highest frictional force. Figure 5 shows the results of the experiments assessing the frictional difference based on fiber composition.

### Testing with skin simulant (Lorica)

**Fig. 6:**
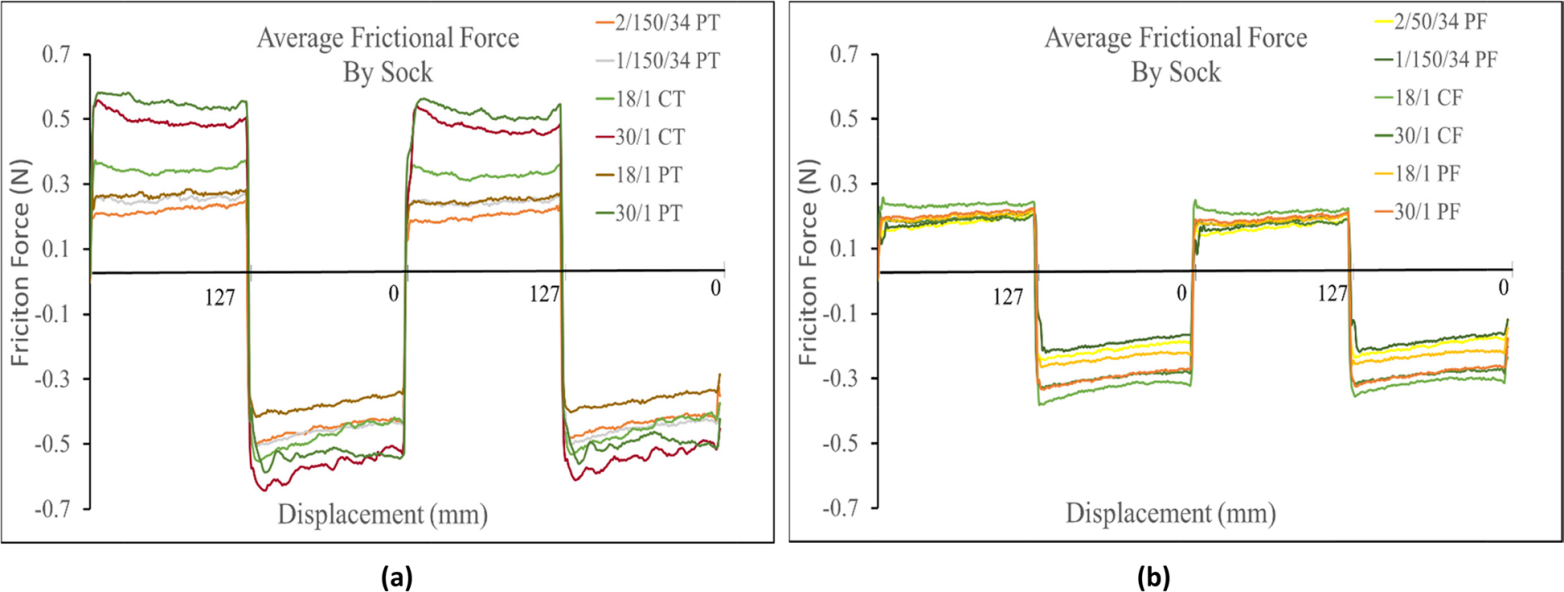
**a** Friction profiles comparing terry knit structures against Lorica skin simulant. **b** Friction profiles comparing single jersey knit structures against the Lorica skin simulant

Against the Lorica, the prominent determining factor in frictional force was again found to be knit structure. The terry knit strucutures were found to have a higher frictional force than their corresponding single jersey knit strucutures (i.e. 30/1 PT > 30/1 PF). This was also seen against the probe. Additionally, the average frictional force seen in all the terry knit structures, in both the positive and negative direction, measured higher than the frictional force in the corresponding single jersey knit structure socks. All single jersey knit strucutures measured similar frictional profiles. (Fig. 6).

**Fig. 7:**
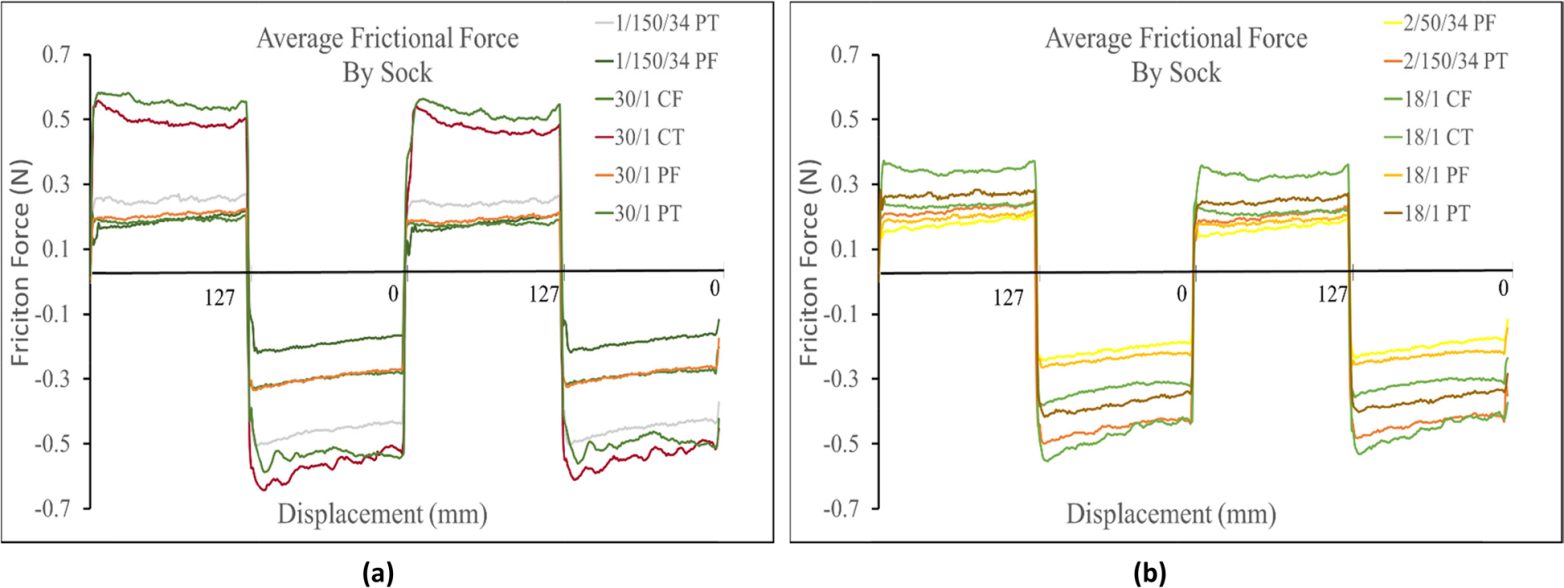
**a** Linear density comparison of terry knit samples against Lorica skin simulant. **b** Linear density comparison of single jersey knit samples against Lorica skin simulant

The linear density again is thought to be a secondary influence on the frictional force of the terry knit socks as the socks made from 30/1 Ne fibers produced a higher frictional force than their corresponding 18/1 Ne socks (Fig. 7b). However, the linear density did not seem to affect the frictional force produced at the interface of single jersey knit samples against the Lorica skin simulant in the positive direction. All four samples were found to have similar friction profiles. In the negative direction the 18/1 cotton single jersey knit registered a higher frictional force than the 30/1 cotton single jersey knit and the 30/1 polyester samples measured a higher frictional force than the 18/1 polyester. For the filament terry knit structures, in the positive direction the 1/150/34 (30/1 Ne equivalent) measured a higher frictional force than the 2/150/34 (18/1 Ne equivalent). In the negative direction the friction profiles were found to be similar. For both single jersey knit filament socks, the friction profiles in both directions were found to be similar.

**Fig. 8:**
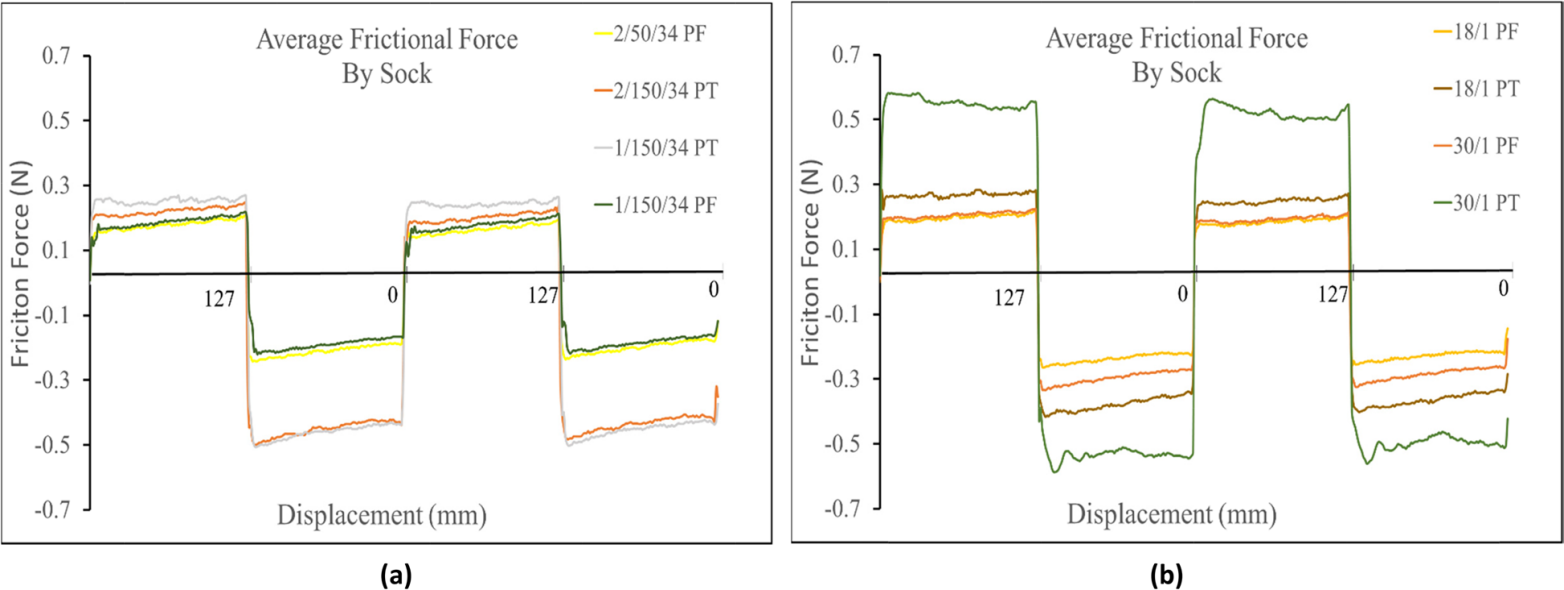
(**a**) Comparison of polyester filament yarn against Lorica skin simulant (**b**) Comparison of spun polyester yarn against the Lorica skin simulant

For samples against the Lorica skin simulant, it was observed that the yarn type does increase frictional force for the terry knit samples. It can be seen in the positive direction that all the spun yarn terry knit samples measured a higher frictional force than their corresponding terry knit filament yarn samples (i.e. 30/1 PT > 1/150/34 PT and 18/1 PT > 2/150/34 PT), as demonstrated in Fig. 8. However, one deviation in trends was observed in the negative direction when measuring the 2/150/34 terry knit samples. From these trials the 2/150/34 terry knit samples registered a slightly higher frictional force than the 18/1 spun yarn sample. The cause of this deviation is unknown and would need to be investigated further.

**Fig. 9:**
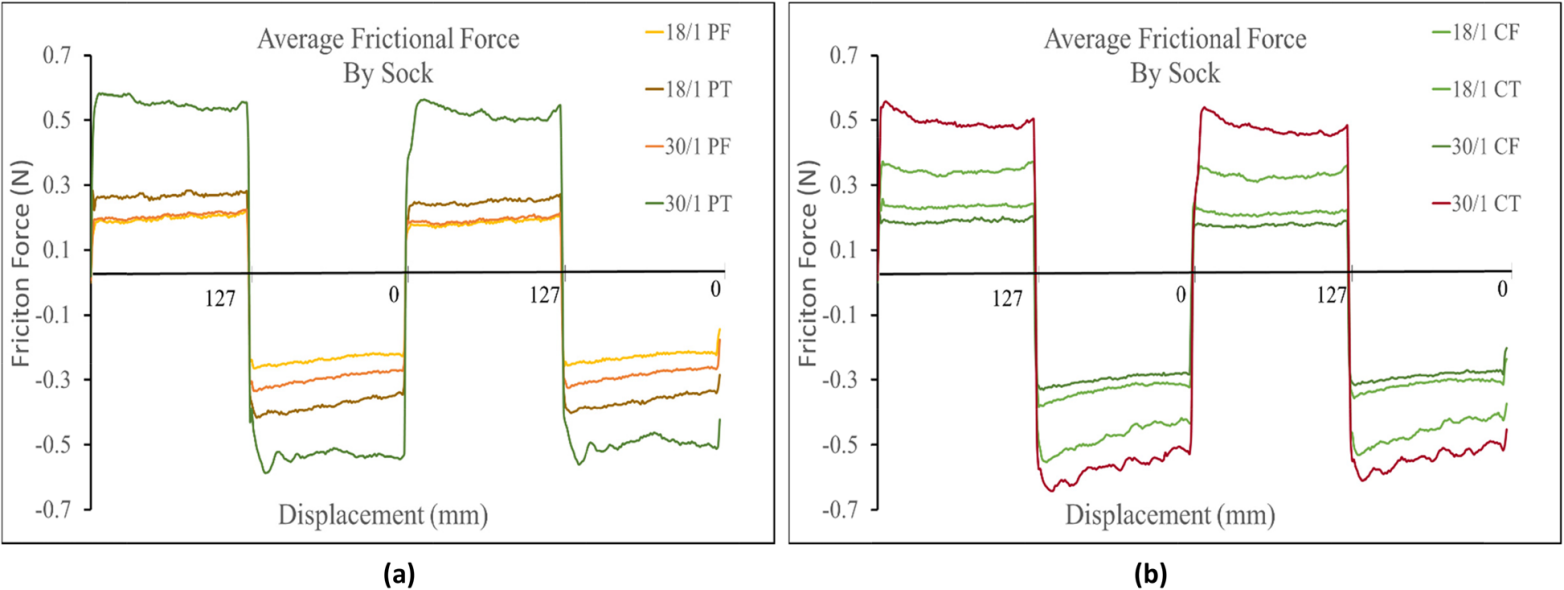
(**a**) Comparison of spun polyester fibers based on corresponding fabric structure, yarn type and linear density against Lorica skin simulant (**b**) Comparison of spun cotton fibers based on corresponding fabric structure, yarn type and linear density against Lorica skin simulant

Against the Lorica, again fiber composition did not play a signifcant role in the frictional force measured. For the terry knit samples the 30/1 polyester measured the highest frictional force, and the 18/1 polyester measured the lowest frictional force in both directions. Once more, all single jersey knit samples measured similar frictional profiles across the socks. In both directions the 18/1 cotton single jersey knit measured the highest frictional force. In the positive direction the lowest frictional force was the 30/1 cotton single jersey knit and in the negative direction it was the 18/1 polyester single jersey knit as shown in Fig. 9.

## Discussion

Based on the market research conducted in this study, it was found that commercially available socks are designed for different end use applications (athletics, dress, everyday use, military, outdoor activities, etc.) and are typically made from cotton, polyester, polyamide, or blends of these fibers. Further, the most common fabric structures found in socks are single jersey and terry knit structures. Socks are also made from both filament and spun yarn, and linear density of fibers can vary between socks. In this current study, the influence of these four different textile parameters was investigated by measuring the friction profile of twelve different sock variations. It was found that, three of the four fabric parameters: knit structure, linear density, and yarn type all affect the friction seen at the sock-skin interface. However, it was determined that fiber composition plays no noticeable role in the friction measured across the bottom inside of a sock.

### Knit structure

Knit structure was found to have the largest influence with regards to measured dynamic frictional force at the sock-skin interface which is consistent with the study completed by Van Amber et al. [3] which found fabric structure to be the dominate mechanism of friction in socks. In Van Amber’s study, terry knit structures also consistently produced a higher frictional force than the single jersey knit structures. However, both these findings partially oppose the findings from a study completed by Baussan et al. [2]. Baussen’s study found that terry knit socks have a lower coefficient of friction than the single jersey knit socks, and thus lower frictional force, in the direction oriented along the terry knit structure. However, Baussen’s study did find that in the direction against the piles (or terries), the terry knit socks produced a higher coefficient of friction than single jersey knit socks. While this current study used a similar method of friction measurement with cyclic motion to that of Baussen’s study, the findings from this current study still corroborate Van Amber’s findings. One possible explanation for why the terry socks produced the higher frictional force is due to the piles that are protruding from the bottom of the sock. These piles are a characteristic of a terry knit structure and are used in socks to provide cushioning for the wearer to stand on. All piles in the socks were the same size however, when these piles compress it causes an increase in the contact angle between the probe/foot and the sock. Increasing the contact angle between the probe/foot at this interface will increase the amount of resistance it takes to slide along the bottom of the sock. Consequently, this would create an increase in the measured frictional force between the sock and foot. Further, because these terries have a preferred orientation (the positive direction), this would explain the sporadic nature seen in the frictional profile when sliding against the terries (the negative direction).

### Yarn linear density

To our knowledge, no previous study found has examined the influence of linear density on the friction measured at the sock-skin interface. In this study it was found that the 30/1 Ne terry knit socks consistently measured higher frictional forces than the 18/1 Ne socks. The likely cause of this is due to the contact area between the probe/Lorica and the terry knit structure. Since the socks made from the 30/1 Ne fibers have a smaller diameter than the 18/1 Ne socks, there are more piles/loops per inch (pile density) to cover the same area. This in turn would mean that there are more fibers in one area resulting in an increased contact area with the probe or lorica for the terry knit socks. The relationship between friction and contact area for textile structures is known as the Howell-Mazur relationship (eq. 1) and is widely accepted as true when measuring dynamic friction at an interface [16]

1 $$F={aR}^n$$

Where F is the frictional force, R is the normal force, a is the coefficient of friction (equal to u only when *n* = 1) and n is the friction index which varies based on material and depends on the materials geometry and its surface roughness. From this relationship we can determine that if pile density is increased, then the geometric contact area is also increased magnifying the effect of measured frictional force at the interface. When examining why the single jersey knit strucutures all have similar friction profiles, one justification would be to assume the lack of piles seen in single jersey knit structure. When there are no piles to increase the potential geometric contact area, the measured friction between the probe and Lorica will be very similar. Even if there are more loops per inch in the 30/1 Ne sock, the increased diameter of the 18/1 Ne sock will counterbalance these. In turn, this means the frictional force values measured in all the single jersey knit structures would be very similar between each sock variation across their entire friction profile which is what was observed across all results.

### Yarn type

Yarn type, for terry knit socks, also played a role in the frictional force experience at the sock-skin interface. Against the probe, all the spun terry knit socks measured a higher frictional force than their corresponding filament socks. While against the Lorica, in the positive direction, again, all the spun terry knit socks produced a higher frictional force than their corresponding filament socks. However, against the Lorica in the negative direction the 2/150/34 filament socks measured the higher frictional force. As for single jersey knit samples, it was seen that yarn type may have a small effect on the friction force experienced. In the positive direction, both the spun single jersey knit samples had similar friction profiles, while in the negative direction the spun samples measured a higher frictional force than the filament samples. Yarn type has previously been studied and it is known that fabrics produced from spun yarn typically measure a higher friction than fabrics made from filament yarn. This is due to the short fibers (yarn hairiness) protruding from the spun yarn surface compared to the smooth surface of the filament yarns. On a microscopic level the short fibers protruding from the spun yarn, interact with the opposing surface increasing the resistance to motion thus increasing the frictional force between the two objects. From this experiment, it is unknown why the 2/150/34 filament yarn measured a higher frictional force in the negative direction than the spun yarn. More analysis would need to be completed to understand this deviation.

### Fiber composition

Fiber composition is (disciplinarily) defined in the textile discipline/industry as the weight percentage of each fiber type that encompasses the textile; this study utilizes socks of 100% Cotton and 100% Polyester fiber composition [17]. As mentioned previously, this study focused on understanding the frictional forces between socks and skin using various sock compositions and the Lorica in-vitro skin simulant only in the dry condition. In the dry state, according to the graphical and statistical analyses conducted, the fiber composition did not significantly affect the frictional force imparted onto the Lorica. This result is supported by existing literature; the study conducted by Van Amber et al. agrees that other factors such as fiber structure, fiber type, and weight had more significant effects on frictional coefficients and values than fiber composition [3]. In addition, because the Coefficient of Friction between polyester and cotton yarn are of similar intensities, frictional force among these two materials will be similar.

Van Amber et al. also suggests that as moisture in the sock-skin interface increases, the frictional force coefficient measured will also significantly change. Hes et al.’s study on how moisture affects friction shows that as the fabric becomes increasingly damp, skin and its underlying layers are increasingly displaced. According to the results of the Hes et al. study, up until the moisture regain value of 40%, increasing moisture in the fabric will increase the friction coefficient measured in the fabric-skin interface. This is because the surface film created at higher moisture levels does not significantly increase the friction coefficient in this environment [18]. This study was conducted solely in a dry environment; thus, the fiber composition did not have a significant effect on frictional force measured between the sock and Lorica skin simulant.

## Conclusion

This parametric design of experiments was completed to clarify and further enhance the understanding of the friction measured at the sock-skin interface. Twelve different sock variations were tested against a plastic probe and Lorica skin simulant to understand how the two interfaces interact with varying sock structures. It was shown that in a dry state, that the knit structure is the most important textile parameter influencing the friction measured at the sock-skin interface. Terry knit structures were found to produce a higher frictional force than single jersey knit strucutures. Further, textile parameters such as linear density and yarn type were found to play secondary roles in the measured friction at sock-skin interface. For socks with terry knit structures decreasing the fiber diameter will increase the frictional force seen in textile materials. Continually, using spun yarn will produce a higher frictional force than using filament yarn. In a dry state fiber composition was not found to influence the frictional force along the sock-skin interface. This study has strategically examined the role of four different textile parameters and has justified exactly how each parameter influences the friction at the sock-skin interface. This knowledge can be used to develop socks that mitigate the risk of friction blisters formation.

## Data Availability

The datasets during and/or analyzed during the current study available from the corresponding author on reasonable request.
